# Changes in physicochemical and biological properties of porcine bone derived hydroxyapatite induced by the incorporation of fluoride

**DOI:** 10.1080/14686996.2016.1263140

**Published:** 2017-02-01

**Authors:** Wei Qiao, Quan Liu, Zhipeng Li, Hanqing Zhang, Zhuofan Chen

**Affiliations:** ^a^ Department of Oral Implantology, Guanghua School of Stomatology, Institute of Stomatological Research, Sun Yat-sen University, Hospital of Stomatology, Guangzhou, PR China; ^b^ Guangdong Provincial Key Laboratory of Stomatology, Sun Yat-sen University, Guangzhou, PR China; ^c^ Zhujiang New Town Dental Clinic, Guanghua School of Stomatology, Institute of Stomatological Research, Sun Yat-sen University, Hospital of Stomatology, Guangzhou, PR China

**Keywords:** Fluoride, porcine bone, biological apatite, physicochemical properties, biological properties, 30 Bio-inspired and biomedical materials, 102 Porous / Nanoporous / Nanostructured materials, 107 Glass and ceramic materials, 211 Scaffold / Tissue engineering / Drug delivery, 302 Crystallization / Heat treatment / Crystal growth

## Abstract

As the main inorganic component of xenogenic bone graft material, bone-derived biological apatite (BAp) has been widely used in implant dentistry, oral and maxillofacial surgery and orthopedics. However, BAp produced via calcination of animal bones still suffers from some drawbacks, such as insufficient mechanical strength and inadequate degradation rate, which impede its application. Fluoride is known to play important roles in both physiological and pathological processes of human hard tissues for its double effects on bones and teeth. In order to understand the effects of fluoride on the properties of BAp, as well as the mechanism behind them, porcine bone derived hydroxyapatite (PHAp) was prepared via thermal treatment, which was then fluoride incorporated at a series concentrations of sodium fluoride, and noted as 0.25-FPHAp, 0.50-FPHAp, and 0.75-FPHAp respectively. The physicochemical characteristics of the materials, including crystal morphology, crystallinity, functional groups, elemental composition, compressive strength, porosity and solubility, were then determined. The biological properties, such as protein adsorption and cell attachment, were also evaluated. It was found that the spheroid-like crystals of PHAp were changed into rod-like after fluoride substitution, resulting in a fluoride concentration-dependent increase in compressive strength, as well as a decreased porosity and solubility of the apatite. However, even though the addition of fluoride was demonstrated to enhance protein adsorption and cell attachment of the materials, the most favorable results were intriguingly achieved in FPHAp with the least fluoride content. Collectively, low level of fluoride incorporation is proposed promising for the modification of clinically used BAp based bone substitute materials, because of its being able to maintain a good balance between physicochemical and biological properties of the apatite.

## Graphical abstract

**Figure:**
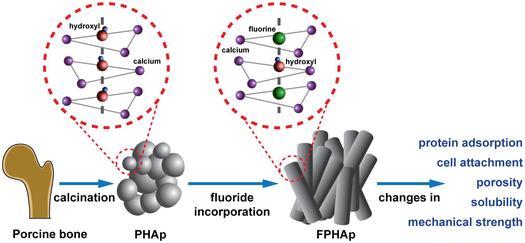

## 1. Introduction

Biological apatite (BAp) is the principal inorganic component of calcified tissues such as bones and teeth. The excellent biocompatibility and osteoconductivity of BAp allow it to be widely used as a substitute material for the reconstruction of osseous defects in dental, craniomaxillofacial and orthopedic surgery. Animal bone derived BAp is known to bear similar chemical composition, porous structure, mechanical performance with human bones. And the dissolution rate of animal bone derived BAp is found to be much closer to the formation rate of human bones [1,2]. There have been a number of studies in recent decades trying to mimic the physicochemical and biological performance of natural bone derived apatite through various modifications on the synthesis of hydroxyapatite (HAp) [3–6]. However, the synthetic methods are either more complex or costly [7], when compared to BAp directly prepared from hard tissues of animals (e.g. bovine bone [8], porcine bone [9] and cuttlefish bone [10]). Among all such sources of BAp, porcine bone appears to show the closest resemblance to human bone in terms of macrostructure and microstructure, chemical composition, and remodeling rate [11], which, together with its abundant supply at a relatively low cost, allow porcine bone derived BAp to be an excellent candidate as a bone graft material. In the augmentation of the alveolar crest and maxillary sinus, favorable bone healing capacity of porcine bone derived BAp has been confirmed [12,13]. However, since thermal treatment at high temperature is involved in most methods during the preparation of porcine bone derived BAp to ensure the elimination of possible pathogens and antigens [9,14,15], the achieved products are often compromised in mechanical strength and biological properties.

Ion substitution is among one of the most widely used methods for the modification of HAp based bone grafting materials, because trace elements (e.g. fluorine, strontium, and zinc) detected in BAp are generally believed to contribute to the physicochemical and biological properties of bone tissue [7,16]. There have been lots of attempts at obtaining apatite materials with better biological performance by incorporating trace ions, such as magnesium [17], strontium [18,19], carbonate [19,20], zinc [21,22], silicon [23,24] and fluoride [10,25]. Fluoride has long been accepted to have direct effects on the stimulation of osteoblasts and mineral apposition in early osteogenesis [26,27]. Fluoride-substituted HAp, where fluoride ions completely or partially replaced hydroxyl groups in HAp, was reported to have a higher crystal growth capability [28] and improved biocompatibility, which might make it a more suitable material for the reconstruction of bone defects [29]. In our previous work, the addition of fluoride in BAp promoted the proliferation and osteogenic activity of osteoblastic-like cells *in vitro* [30]. However, the previous study focused on the effects of released fluoride ions from fluorinated BAp, rather than on a systematic evaluation of the physicochemical properties and of the relevant biological properties of the materials, which was of interest in the current study.

The aim of this study was to provide a simple and cost-effective way to modify the clinically used xenogenic bone graft materials, and reveal the underlying mechanism. PHAp was prepared from porcine cancellous bone with an immersion-calcination process as reported previously [9], and then fluoride substituted at a series of concentrations. The physicochemical characteristics of the materials, including crystal morphology, elemental composition, crystallinity, functional groups, compressive strength, porosity and solubility, were systematically studied. The protein adsorption and cell attachment, which are both closely related to the surface topography of materials, were also evaluated *in vitro*. Specifically, due to both the complexity in the dissolution of fluoride containing calcium phosphate and some methodological drawbacks as discussed in our earlier studies [31,32], it has been realized that the traditional ‘excess-solid’ approach is inappropriate for determining the solubility of materials in this work. Therefore, the solid titration method, which depends on crystal nucleation near the solution equilibrium that has been established to be more reliable and reproducible in our previous work [32–35], was used to determine the solubility of PHAp and FPHAp in the present study.

## 2. Materials and methods

### 2.1. Sample preparation

PHAp was prepared by simple chemical and thermal treatments as previously reported [9]. Briefly, cancellous bone harvested from porcine femoral epiphysis was boiled in distilled water (2 h) for degreasing and easier removal of soft tissue like periosteum and bone marrow. Then the bones were dissected into regular blocks (5 mm^3^) with cut-off machines (Accutom-50, Struers, Ballerup, Denmark) coupled with cooling water, calcinated to 800°C at a heating rate of 10°C min^–1^ and held at this temperature for 2 h in air in a muffle furnace (SGM6812BK, XIGEMA, Xi’an, China). The thermal treatment was followed by a thoroughly cleaning process in deionized water (Milli-Q, Millipore, Billerica, MA, USA) to remove the organic ashes and other mixed impurities within the macropores of the bone blocks. Then the samples were randomly classified into four groups, and immersed in deionized water (as control) and sodium fluoride solution (NaF, analytical grade, Guangzhou Chemical Reagent Factory, Guangzhou, China) in a series of concentrations (0.25, 0.50, and 0.75 mol l^–1^) for 24 h, respectively. Afterwards, calcination was performed again at 700°C in air (heating rate: 10°C min^–1^, holding time: 3 h). The annealed samples, known as PHAp, 0.25-FPHAp, 0.50-FPHAp and 0.75-FPHAp, were rinsed with deionized water thoroughly and dried at 80°C overnight. The prepared samples were stored in a desiccator over silica gel before use.

### 2.2. Sample characterizations

The crystal morphology of all samples was examined using transmission electron microscopy (TEM, Tecnai G2 F30, FEI, Eindhoven, the Netherlands) and scanning electron microscopy (SEM, Quanta 400 FEG, FEI). For TEM, powdered samples were ultrasonically dispersed in ethanol, and examined in bright field mode using an accelerating voltage of 120 kV. Selected area electron diffraction (SAED) patterns were obtained for each sample. The elemental composition and distribution of each sample were examined by energy-dispersive X-ray spectroscopy (EDS, SU1510, Hitachi, Tokyo, Japan) and SEM-EDS mapping. The samples were cemented on copper stubs using graphite adhesive and scanned at 15 kV. For the observation of crystal morphology at high magnification, the samples were sputtered with gold (20 s, SCD 005, BAL-TEC, Balzers, Liechtenstein) before observation.

The functional groups of PHAp and FPHAps were identified using Fourier-transform infrared spectroscopy (FTIR, Vector 33, Bruker Optics, Ettlingen, Germany). Powdered samples were mixed with pre-dried KBr powder (1:100 by mass; IR grade, Merck, Giessen, Germany), and then uniaxially compressed into pellets for examination (10 MPa). IR spectra were collected in transmittance mode with a scanning range of 4000–400 cm^−1^ at a 0.2 cm^−1^ resolution. Crystal characteristics of PHAp and FPHAp were examined using X-ray diffractometer (XRD, Empyrean, Panalytical, Eindhoven, the Netherlands). Powdered samples were mounted on glass stubs. A diffracted beam graphite monochromator was used to produce copper Kα1 radiation with a wavelength of 1.54056 Å. A scanning speed of 10° (2θ) min^–1^ and a step size of 0.01° were adopted over a 2θ range of 20–60 °. Stoichiometric HAp pattern (JCPDS card #09-0432) and FAp pattern (JCPDS card #15-0876) were used as references. Apatite lattice parameters (hexagonal system) were calculated in software (MDI Jade, v. 6.1, Materials Data, Livermore, CA, USA) by using the correlation between interplanar distances and the Miller indices of reflecting plane h, k. The *a*- and *c*-axis dimensions were determined from the (3 0 0) and (0 0 2) planes, respectively. The crystallinity was evaluated by the relation between the intensity of (3 0 0) reflection and the intensity of the hollow between (1 1 2) and (3 0 0) reflections [36].

### 2.3. Mechanical strength determination

For mechanical evaluation, the regular blocks (5 × 5 × 5 mm^3^ cubes) of PHAp, 0.25-FPHAp, 0.50-FPHAp and 0.75-FPHAp were mounted to Universal Testing Machine (E3000, Instron, Norwood, MA, USA) for uniaxial compression tests. In brief, the tests were performed in air, at a constant cross-head speed of 0.5 mm min^–1^. At least 20 samples in each group were tested. The compressive strength was estimated according to the cross-sectional area and the applied maximum force when the block was crashed.

### 2.4. Porosity determination

The porosity of the samples was assessed using mercury intrusion porosimetry (AutoPore IV 9500, Micromeritics, Norcross, GA, USA). Approximately 0.5 g sample was analyzed in a pressure range of 0.51–59900 psia (corresponding to the pore diameters from approximately 350 μm to 0.003 μm). Representative mercury intrusion data were used for the plot of pore diameter vs*.* cumulative intrusion and incremental intrusion in software (OriginPro, v.9.0, OriginLab, Northampton, MA, USA).

### 2.5. Solubility determination

The solid titration method, which has been extensively used for the determination of the solubility of calcium phosphate based materials [32,34,35,37,38], was adopted in this study. In brief, the samples to be determined were ground into fine powder manually using an agate pestle and mortar, and passed through a 200-mesh sieve (0.075 mm). Synthetic HAp used as a reference and for the calibration of the system was prepared using a standard precipitation method [39]. The wide-neck borosilicate reaction flask for the titration was maintained in 37.0 ± 0.1°C water bath, shielded and flushed with pure nitrogen (99.999%, Foshan Oxygen, Guangzhou, China), and sterilized with ultraviolet lamps (Spectroline E14/F, Spectronic, Westbury, NY, USA). 100 mmol l^–1^ potassium chloride (KCl, ARISTAR, BDH, Poole, UK) was used as the background solution. Specifically, to avoid the reaction between the released fluoride ions and the glass flask by the formation of tetrafluorosilane (SiF_4_), the inner surface of the flask was thoroughly coated with a thin layer of paraffin wax (BDH, UK) as described elsewhere [40].

The process of titration was monitored by a semiconductor-diode laser beam (1 mW CW, 194-010, RS Components, Corby, UK) and a laser detector recording the scattering of laser caused by the presence of undissolved solid. This method is based on detecting the point at which no further solid dissolves, or a new precipitate forms, via small increments of solid that must dissolve completely before a further increment is added. When the end-point was reached, the pH value was then adjusted downward (from 0.5 to 2 units) by adding 1 mol l^–1^ hydrochloric acid (HCl, analytical grade, Guangzhou Chemical Reagent Factory, Guangzhou, China) until all the particles had dissolved (indicated by the laser signal back to the baseline and stable pH of the solution). Then, the next run of titration could be continued. The end-points at different pH values obtained by repeating this procedure were used to plot the solubility isotherm in software (OriginPro, v.9.0).

### 2.6. Cell attachment assays

Human osteoblast-like cells MG63 (human osteosarcoma cell line), a well-established osteoblastic-like cell line to assess the cytocompatibility of biomaterials, was used for cell attachment assay. MG63 cells were purchased from cell banks of the Chinese Academy of Science (Shanghai, China), cultured in Dulbecco’s modified Eagle’s medium (DMEM, Hyclone, South Logan, UT, USA) supplemented with 10% (vol/vol) fetal bovine serum (Gibco, Tulsa, OK, USA) and 100 U ml^–1^ penicillin-streptomycin (Gibco) at 37°C in a humidified atmosphere with 5% CO_2_. PHAp and FPHAp blocks were sterilized by irradiation with gamma rays at a dose of 25 kGy before usage. The MG63 cells were seeded on the porous blocks at a density of 1 × 10^5^ cells per block. After 24 h, the blocks were thoroughly rinsed with phosphate buffered saline three times, and fixed with 2.5% glutaraldehyde at 4°C overnight. Then, the samples were dehydrated with gradient alcohols and dried with CO_2_ using a critical point dryer (HCP-2, Hitachi). Finally, the samples were coated with a gold sputter (E1010, Hitachi ion sputter) before SEM observation (Quanta 400 FEG, FEI).

Quantitative evaluation of cell attachment was performed by cell counting kit-8 (CCK-8, Dojindo, Kumamoto, Japan). In brief, 24 h after the seeding of cells, CCK-8 solution was added to the culture medium at a ratio of 1:10, mixed well and allowed to incubate for 2 h. Then, 100 μl of the supernatant was collected and transferred to 96-well plate for the measurement of colorimetric change using microplate spectrophotometer (Infinite M200, Tecan, Männedorf, Switzerland) at the wavelength of 450 nm with a reference wavelength of 650 nm. Cell attachment was evaluated by comparing the rise in optical density value of the FPHAp groups to PHAp.

### 2.7. Protein adsorption assays

For the evaluation of protein absorption capacity of the apatite, PHAp, 0.25-FPHAp, 0.50-FPHAp and 0.75-FPHAp were respectively placed in centrifuge tubes and soaked with 1 mg ml^–1^ BSA standard solution (ThermoFisher Scientific, Hudson, NH, USA) at a solid/liquid ratio of 100 mg ml^–1^. Tubes containing 1 mg ml^–1^ BSA solution but without any sample was set as a control. After the incubation on a shaker at 37°C for 120 min, the supernatant was collected by centrifuge, and transferred to a new 96-well plate for the measurement of the remaining protein content using BCA Protein Assay Kit (ThermoFisher Scientific). The absorption of BSA was calculated by the reduction in optical density value of each group compared with the control.

### 2.8. Statistical analysis

For results of compressive strength test, cell attachment assay and protein adsorption assay, the data were expressed as means ± standard deviations (SD). One-way analysis of variance (ANOVA) was performed with SPSS (v.13.0, IBM SPSS, Chicago, IL, USA), and the level of significant difference was defined and noted as * *p*<0.05.

## 3. Results

### 3.1. Sample characterization

**Figure 1.**
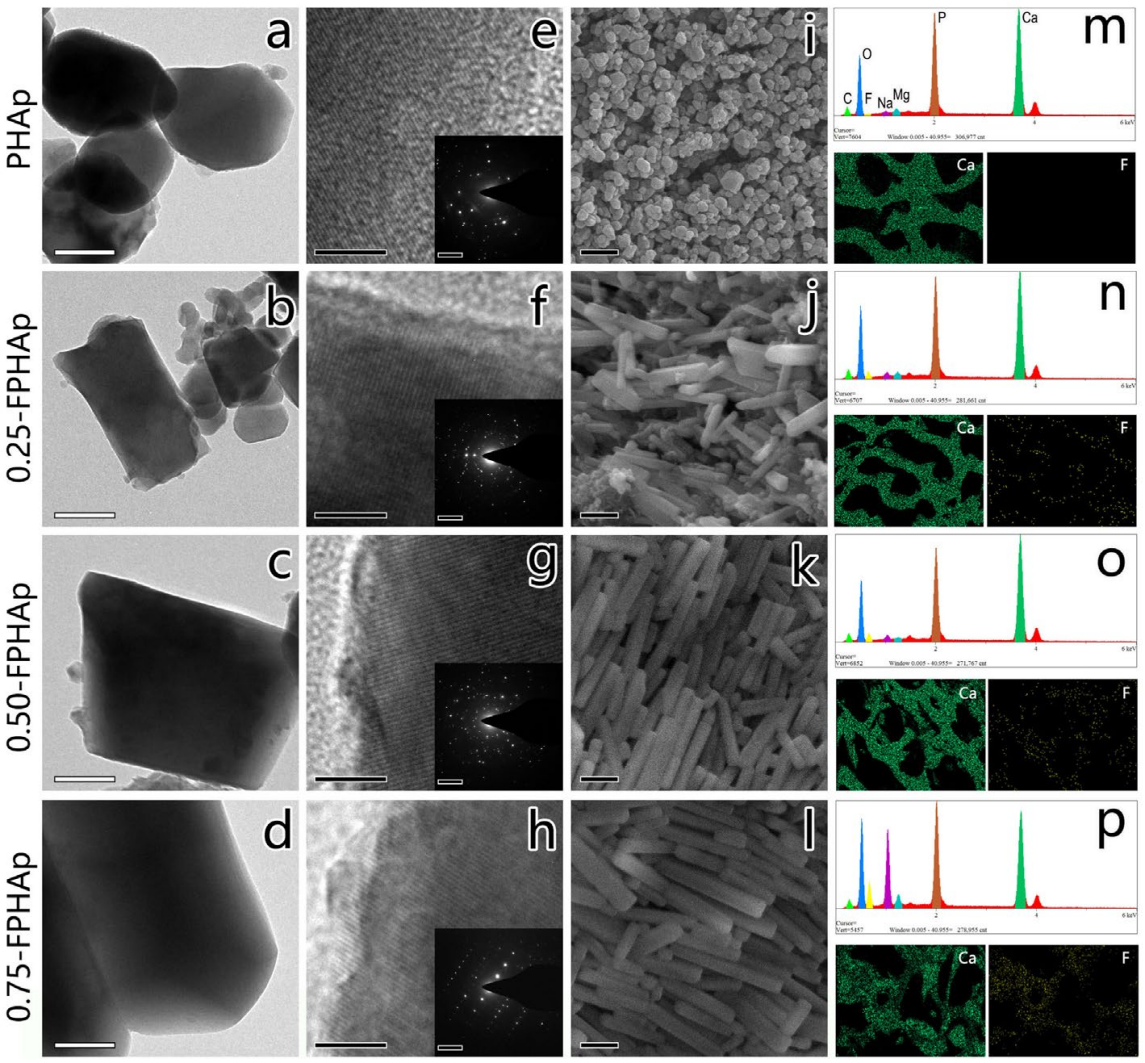
TEM images (scale bar = 100 nm) and magnified section (scale bar = 5 nm) with corresponding SAED patterns (scale bar = 5 1/nm) of (a, e) PHAp; (b, f) 0.25-FPHAp; (c, g) 0.50-FPHAp; (d, h) 0.75-FPHAp. SEM images (scale bar = 1 μm) of (i) PHAp; (j) 0.25-FPHAp; (k) 0.50-FPHAp; (l) 0.75-FPHAp. EDS spectra, as well as the distribution of calcium and fluoride by EDS mapping of (m) PHAp; (n) 0.25-FPHAp; (o) 0.50-FPHAp; (p) 0.75-FPHAp.

Representative crystals for PHAp, 0.25-FPHAp, 0.50-FPHAp and 0.75-FPHAp are shown in Figure 1: TEM in Figure 1(a)–(d); SEM in Figure 1(i)–(l); and corresponding SAED patterns in Figure 1(e)–(h). The PHAp crystals exhibited a spheroid shape with a diameter around 200 nm, while rod-like crystals appeared after the incorporation of fluoride. For 0.25-FPHAp, both spheroid- and rod-like shaped crystals were observed, ranging from ~100 nm to ~10 μm. In contrast, the crystals of 0.50-FPHAp and 0.75-FPHAp became more homogeneous in both size and shape (i.e. rod-like with length longer than 400 nm). Additionally, the SAED analysis showed that FPHAp had typical apatitic structure significantly different from PHAp. SEM mapping (Figure 1(m)–(p)) showed that fluorine was evenly distributed in the porous structure. Moreover, the EDS results as demonstrated in Table 1 revealed the major chemical elements of PHAp and FPHAps to be calcium, phosphorus, oxygen, and carbon, though magnesium and sodium can also be detected at a relative low level. The content of fluorine in FPHAp was correlated with the concentration of sodium fluoride used in their preparation.

**Table 1.** Elemental analysis of PHAp and FPHAp samples by EDS.

	Atomic percentage (wt. %)						
	Ca	P	O	C	Na	Mg	F
PHAp	44.6 ± 0.4	20.6 ± 0.3	27.8 ± 0.4	3.3 ± 0.7	1.3 ± 0.5	1.8 ± 0.2	0.6 ± 0.9
0.25-FPHAp	42.5 ± 0.1	19.4 ± 0.4	27.6 ± 0.8	3.6 ± 0.3	2.0 ± 0.3	1.4 ± 0.1	3.5 ± 0.1
0.50-FPHAp	43.9 ± 0.1	19.2 ± 0.1	26.1 ± 0.3	3.4 ± 0.2	2.1 ± 0.1	1.10 ± 0.02	4.2 ± 0.1
0.75-FPHAp	40 ± 2	18.8 ± 0.1	24.3 ± 0.6	3.3 ± 0.1	4.3 ± 2.2	1.8 ± 0.3	7.2 ± 0.6

**Figure 2.**
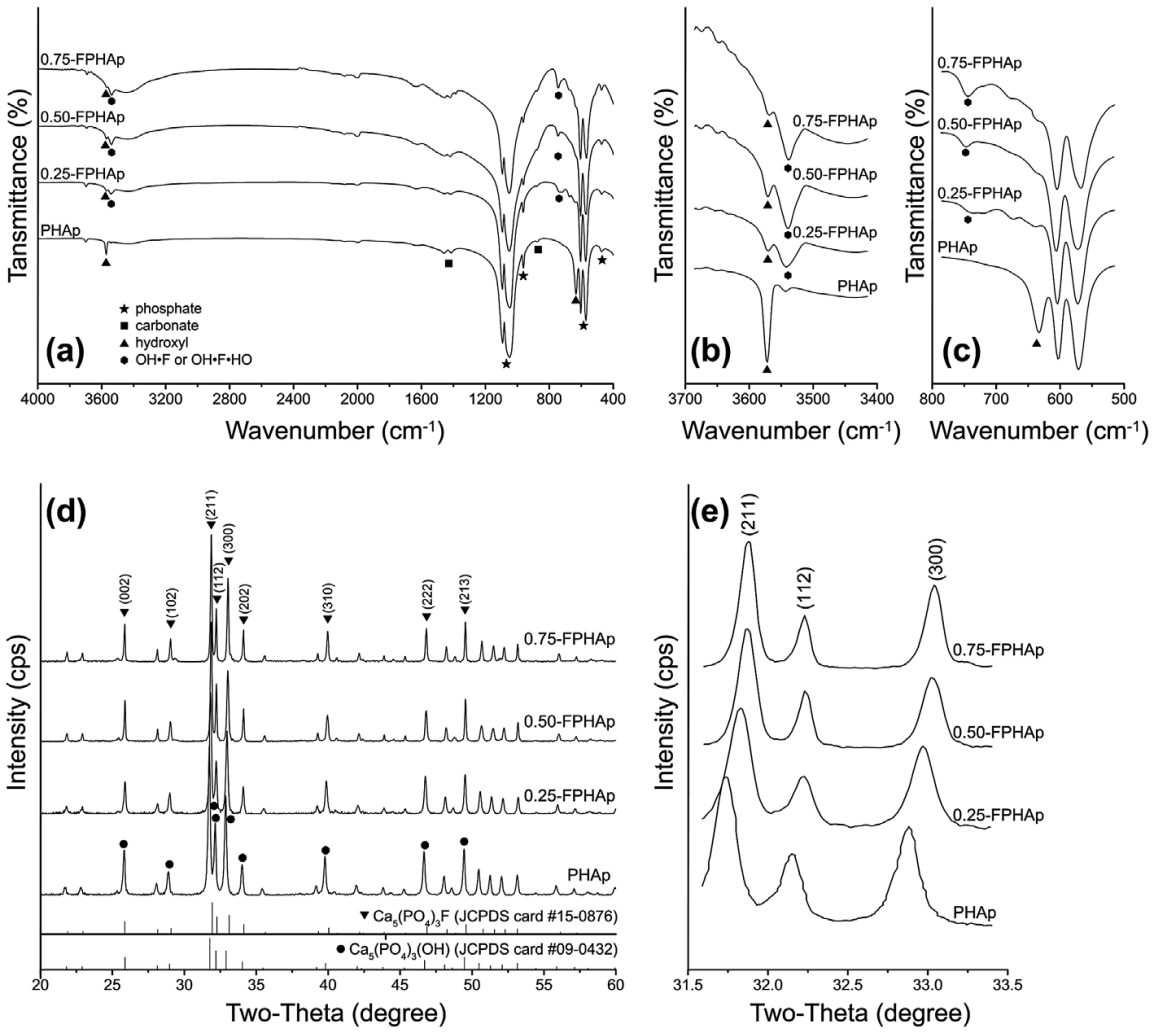
(a) FTIR spectra, (b) expanded water stretching region and (c) OH libration region of PHAp, 0.25-FPHAp, 0.50-FPHAp, and 0.75-FPHAp. (d) XRD patterns and (e) expanded X-ray reflections of PHAp, 0.25-FPHAp, 0.50-FPHAp, and 0.75-FPHAp (all spectra are vertically offset for clarity).

In FTIR spectra, characteristic functional groups of phosphate, hydroxyl and carbonate groups for both PHAp and FPHAp were observed (Figure 2(a)). P-O *ν*1 (962 cm^−1^), *ν*2 (473 cm^−1^), *ν*3 (1092 and 1046 cm^−1^) and *ν*4 (602 and 568 cm^−1^) vibrational bands remained constant for all samples. In addition, C-O *ν*2 (874 cm^−1^) and *ν*3 (1461–1410 cm^−1^) vibrational bands were found in both PHAp and FPHAps. It can also be noted that with the increase of fluoridation, the OH stretching band at 3572 cm^−1^ that was quite evident in PHAp became weaker, while the band at 3538 cm^−1^, pertaining to OH-F or OH-F-HO, exhibited a gradual increase (Figure 2(b)). Moreover, the OH libration band shifted from 634 cm^−1^ in PHAp to 745 cm^−1^ in 0.75-FPHAp (Figure 2(c)).

XRD patterns of PHAp (Figure 2(d)) were found to be consistent with that of the stoichiometric HAp (JCPDS card #09-0432), which, with the increase of fluoridation, gradually shifted to a higher-angle direction and fitted better with that of FAp (JCPDS card #15-0876; Figure 2(e)). This shifting was shown in the enlargement of 31.5–33.5 ° (2θ) section (Figure 2(d)), i.e. the (2 1 1), (1 1 2) and (3 0 0) peaks shifted evidently to the higher-angle direction. Besides, the diffraction peaks of FPHAp also became sharper with the increase in the fluoride content, indicating an increasing crystallinity, which was confirmed by the calculated crystallinity shown in Table 2. Meanwhile, the *a* lattice constant of PHAp was a little lower than that of stoichiometric HAp (*α* = 0.9432 nm), which became even lower with the substitution of fluoride. However, there was no discernible difference in the *c* lattice constant among samples.

**Table 2.** Lattice constants and crystallinity of PHAp, 0.25-FPHAp, 0.50-FPHAp, and 0.75-FPHAp. With the rise in fluoride content, the crystallinity of the apatite steadily increased. The incorporation of fluoride also resulted in significant decline of *a* lattice constant of the apatite, while the c lattice constant did not show any discernable difference.

	Lattice constant (Å)		Crystallinity(%)
	*a*-axis	*c*-axis	
PHAp	9.42541	6.88685	94.4 ± 1.6
0.25-FPHAp	9.40576	6.88174	96.9 ± 2.2
0.50-FPHAp	9.38676	6.88120	98.7 ± 2.2
0.75-FPHAp	9.38234	6.88467	98.7 ± 1.4

### 3.2. Mechanical strength

**Figure 3.**
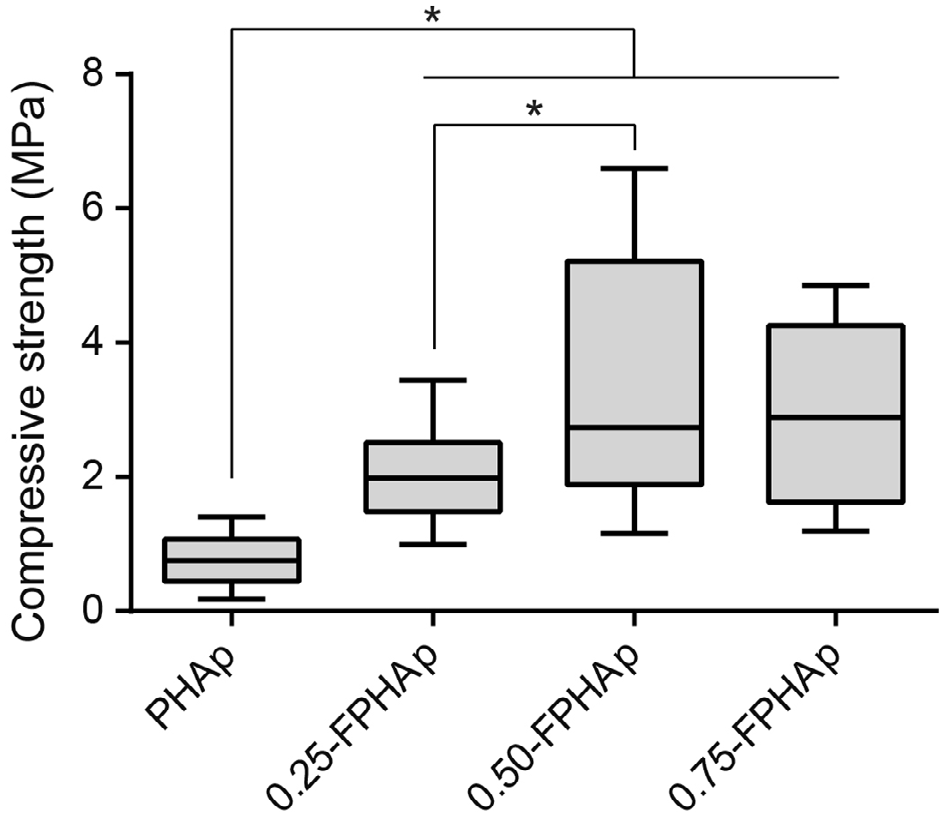
Box plot showing the compressive strength of PHAp, 0.25-FPHAp, 0.50-FPHAp, and 0.75-FPHAp.

Figure 3 shows that the incorporation of fluoride groups has significantly increased the compressive strength of PHAp, which was lower than 1 MPa. Moreover, the mechanical strength of 0.50-FPHAp was higher than that of 0.25-FPHAp with significant difference (*p*<0.001). However, 0.75-FPHAp, with more fluoride substitution, did not show better mechanical performance than 0.50-FPHAp.

### 3.3. Porosity

**Figure 4.**
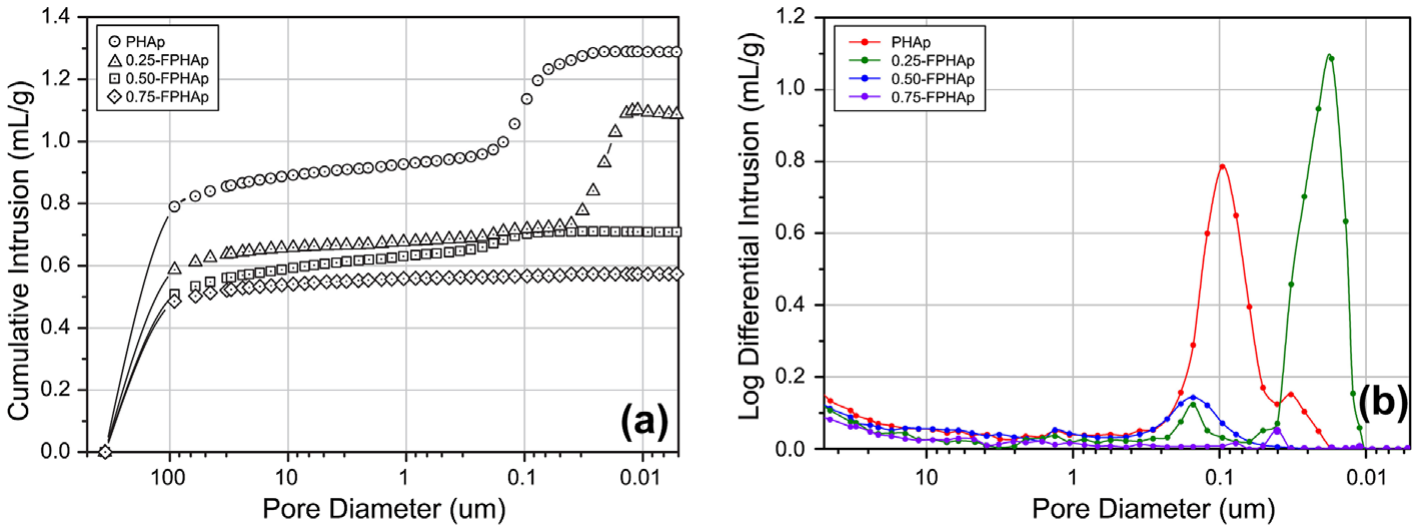
(a) Mercury intrusion cumulative curves of PHAp, 0.25-FPHAp, 0.50-FPHAp, and 0.75-FPHAp. (b) Incremental intrusion in relate to pore diameter showing the pore diameter distribution of PHAp, 0.25-FPHAp, 0.50-FPHAp, and 0.75-FPHAp.

Mercury intrusion porosimetry curves were shown as cumulative intrusion (Figure 4(a)) and incremental intrusion (Figure 4(b)), in relation to the pore diameter. Followed by the considerable intrusion in pores between ~400 μm and ~50 μm, which indicated the interstices spaces, the mercury penetration in small pores within the materials was significantly attenuated. For PHAp, there was a significant intrusion detected in the interval from 0.2 to 0.03 μm, while no further intrusion was seen afterwards. Another significant intrusion was detected in 0.25-FPHAp, but it was distinctly in the diameter between 0.06 and 0.01 μm. However, for 0.50-FPHAp, only a small intrusion was detected in the range between 0.3 and 0.1 μm, and there was almost no further intrusion after 50 μm in 0.75-FPHAp. Moreover, the results of mercury intrusion porosimetry also provided the total intruded volume and the calculated porosity values for each sample, indicating the porosity of PHAp, 0.25-FPHAp, 0.50-FPHAp and 0.75-FPHAp to be around 79, 71, 67 and 62%, respectively.

### 3.4. Solubility

**Figure 5.**
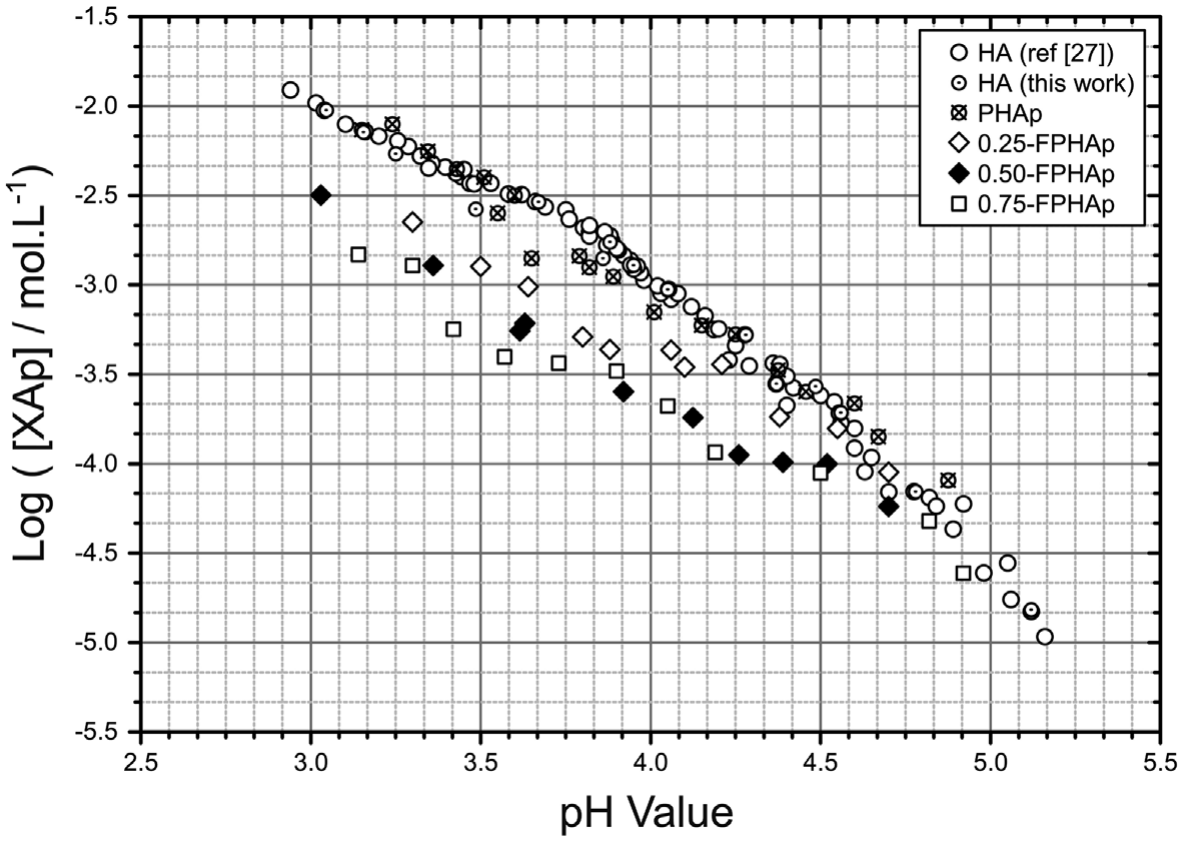
Solubility isotherms of PHAp, 0.25-FPHAp, 0.50-FPHAp, and 0.75-FPHAp 100 mM KCl solution at 37.0 ± 0.1°C by solid titration. Solubility data of stoichiometric HAp were shown for comparison.

As shown in Figure 5, the solubility isotherm of as-prepared HAp in this study was perfectly in consistent with that in our previous work. The solubility isotherms of PHAp and synthetic HAp were almost overlapped to each other, suggesting their solubility to be quite similar over the pH range of 3.0–5.0, except for a marginal difference at pH 3.5–4.0. Moreover, it is also evident that with the increase in fluoride content, the location of the solubility isotherms of FPHAps became lower, which suggested their solubility decrease with the rise of fluoride incorporation, especially at the low pH range (i.e. 3.0–4.0).

### 3.5. Cell attachment

**Figure 6.**
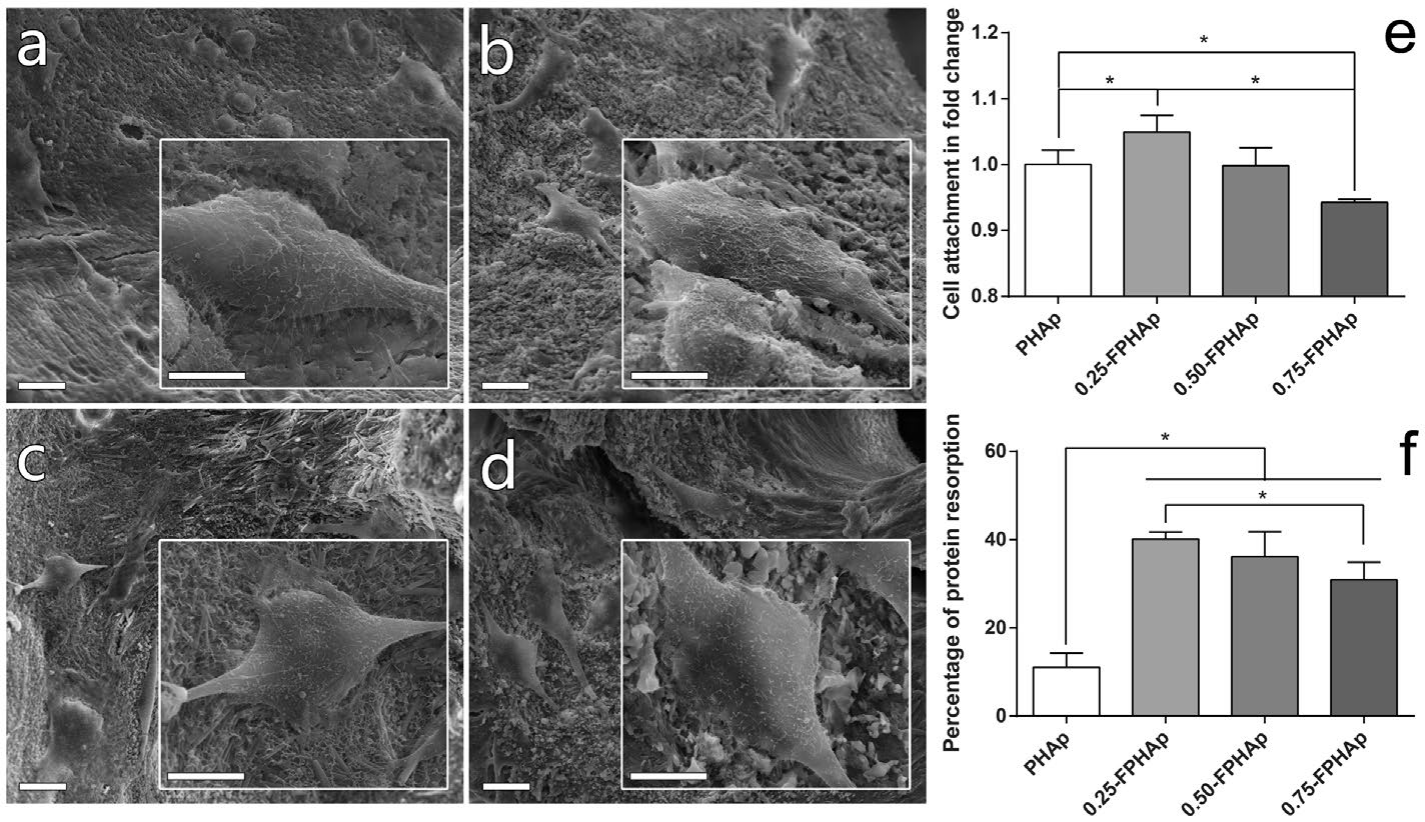
SEM images (scale bar = 20 μm) and inserted magnified view (scale bar = 10 μm) showing the adhesion of human osteoblastic-like MG63 cell on the surfaces of (a) PHAp, (b) 0.25-FPHAp, (c) 0.50-FPHAp, and (d) 0.75-FPHAp. (e) Quantitative evaluation of cell attachment showed only 0.25-FPHAp favored increased attachment of MG63, while 0.75-FPHAp was inferior than PHAp for cell attachment. (f) Protein absorption assay demonstrated that the incorporation of fluoride resulted in significant higher protein absorption of the apatite.

As shown in Figure 6(a)–(d), after a culture time of one day, the cells were firmly attached to the surface of the PHAp, 0.25-FPHAp, 0.50-FPHAp, and 0.75-FPHAp. In the inserted high magnification view, it can be seen that the cells appeared to be polygonal in shape with many lamellipodia and filopodia extensions. Then cell attachment on the surface of apatite was also quantified by CCK8 assays one day after seeding, as shown in Figure 6(e). There were significantly (*p* < 0.05) more attached cells on the surface of 0.25-FPHAp, compared with the other groups. However, the effect seemed not fluoride concentration dependent, because the cell attachment on 0.75-FPHAp was the lowest among the four tested samples.

### 3.6. Protein adsorption and cell attachment

As shown in Figure 6(f), the protein adsorption in fluoride incorporated groups was significantly higher (threefold to fourfold increase) compared with PHAp. However, interestingly, the protein adsorption on 0.75-FPHAp was lower than that on 0.25-FPHAp, although the former had higher fluoride content.

## 4. Discussion

### 4.1. Characterization of PHAp/FPHAp

The *in vivo* crystals of bone-derived BAp have been found to be irregular platelets, smaller than 100 nm [41, 42], which are substantially distinct from PHAp obtained in the present study (Figure 1(a), 1(e) and 1(i)). In addition, BAp has conventionally been recognized as an AB-type carbonated hydroxyapatite [42,43], while PHAp in this study was found to be a B-type, i.e. phosphate groups were partially substituted by carbonate [44] (Figure 2(a)). Moreover, the carbonate content in the prepared sample was significantly lower than that in the raw materials (i.e. porcine bone). All of these indicated that thermal treatments involving high temperature may have dramatic effects on the morphology, crystal structure and composition of BAp, as extensively discussed elsewhere [42]. It was reported that more carbonate can be maintained by decreasing the temperature of heat treatment or involving CO_2_ atmosphere during calcination;[42,45] however, conventional thermal treatment, as adopted in this study, is still recommended to avoid the residual of organic components, which might result in undesirable host response [7].

Fluoride has been well recognized for its potential behavior in promoting mineralization and crystallization of calcium phosphates in the formation of bone, enamel [28,46] as well as synthetic apatite [47]. However, studies concerning the effects of fluoride incorporation on the physicochemical and biological properties of BAp are rare [9,10,25]. Additionally, accurate measurement of crystal parameters in BAp samples was difficult due to the overlapping of crystals in SEM, as well as breakage of crystals during sample preparation in TEM. In the present study, the influence of fluoride addition on the crystal shape, size and crystallinity of porcine bone derived BAp was confirmed simultaneously by TEM, SEM (Figure 1) and XRD (Figure 2(d) and 2(e); Table 1), and the significant changes of the materials after the fluoride incorporation corresponded to the previous reports on synthetic fluoride-containing calcium phosphates [47–49]. Since we detected the effects of high level of fluoride incorporation on the morphology of apatite crystals [9], this work has further confirmed the ability of fluoride to promote the ordered assembly of rod-like crystals, even at much lower concentrations. However, it should also be noted that at low fluoride groups (especially 0.25-FPHAp), the shape of crystals was actually a mixture of spheroid-like and rod-like, with more varied sizes (Figure 1).

The incorporation of fluoride into HAp has been found to form fluoride-hydroxyl bonds (OH·F·OH and OH·F), as the disordered hydroxyl groups within HAp are the favored sites for fluoride substitution [50,51]. This was confirmed by the shifting of the whole XRD patterns to higher-angle direction in this study, i.e. from HAp to FAp (Figure 2(d) and 2(e)), as well as the shifted peaks from hydroxyl groups to fluoride-hydroxyl bonds (Figure 2(b) and 2(c)). Moreover, there was no significant change to lattice constant *c* by this fluoride substitution, because the fluoride-hydroxyl substitution occurred in the channel along the *c*-axis, while the contraction in *a*-axis may be due to the slightly smaller size of F^-^ (136 pm) compared with OH^-^ (153 pm) [44,49,52].

### 4.2. Changes in mechanical and biological properties

Compressive strength is a key factor for bone substitute materials, especially when being used in the load-bearing areas. The mechanical properties of porcine bone are reported to be the most similar to that of human’s compared with the other animals [53]. However, the calcination process dramatically decreases the mechanical performance of the calcium phosphate based bone substitute, making it a potential risk for the long-term outcome after bone augmentation surgery [54]. It has been reported that the addition of fluoride in the sintering could alter the densification behavior of HAp, which leads to enhanced mechanical performance [51]. In this study, the incorporation of fluoride was found to increase the compressive strength of the apatite. This effect was evident in the lowest fluoride content group (i.e. 0.25-FPHAp), and became more prominent in 0.50-FPHAp (see Figure 3). However, further increase in fluoride level in 0.75-FPHAp barely contributed to a significant increase in compressive strength compared with 0.50-FPHAp, implying the effect of fluoride incorporation on the mechanical strength may have reached its limit at this fluoride level.

In terms of the porosity of PHAp and FPHAp, it was found that even after the calcination process, PHAp managed to maintain the micropores approximately 0.1 μm in diameter (Figure 4(b)), which seemed to correspond to lacunae-canaliculi spaces, where osteocytes locate and communicate with each other within the bone matrix [54]. However, the porosity of fluoride incorporated samples is significantly different, which can be associated with the quick growth of crystals caused by the addition of fluoride during the calcination. To be specific, in the low fluoride incorporated group (i.e. 0.25-FPHAp), the incomplete and non-homogeneous crystal growth only resulted in the reduction of the pore diameter to 0.01–0.06 μm. In contrast, when the well assembled rod-like crystals are formed in the higher fluoride groups (i.e. 0.50-FPHAp and 0.75-FPHAp), the pores smaller than 1 μm almost vanished. Although the mercury intrusion porosimetry also demonstrated the porosity to be negatively related to the fluoride content of the material, we propose the values should only serve as a reference, because they might be biased by the interparticle voids, rather than the internal pores of the samples. Moreover, since it was reported that any pore present in the scaffold can lead to reduced strength and stiffness of the material [55], the changes in porosity of the samples can also be responsible for the distinct mechanical performance mentioned above.

However, when regarding the optimal porosity for bone substitute materials, there must be a balance between the mechanical properties and the biological properties of the materials. This is because both macropores and micropores on bone substitute materials are essential for bone regeneration due to their roles in the attachment of osteogenic cells, the formation of blood vessels, as well as the transportation of nutrition and waste [55,56]. In addition, the presence of micropores can increase the specific surface area for protein adsorption and the subsequent adhesion of bone forming cells [57]. Our data indicated that in the high fluoride containing FPHAp, though the loss of micro/nano-pores contributed to the better compressive strength of the material, the protein adsorption and cell attachment decreased significantly compared with the low fluoride containing FPHAp. Moreover, we noticed that there remained some differences in cell morphology among different groups of materials. To be specific, on the surface of low fluoride containing materials, more cells were well stretched, with extensions of lamellipodia and filopodia, which indicates them to be tight in adhesion and active in migration. Meanwhile, on the surface of 0.75-FPHAp, the spreading of the cells was mostly inferior to the other groups.

Nevertheless, even though PHAp was found to be more porous compared with FPHAp, its biological performance was intriguingly inferior to 0.25-FPHAp. Moreover, there were more cells in a flattened fried egg shape on PHAp, suggesting them to be less active in migration. Possible explanation for the phenomenon is that the biological properties of the apatite can be affected by some other factors, such as surface topography, surface charge, ions release, and wettability [57]*.* For instance, it was found that the adhesion and proliferation of human mesenchymal stem cells on HAp was dependent upon the grain size of the substrate [58], specifically, the HAp with 200 nm or smaller grain size could significantly reduce the cell attachment, when compared with the 1500 nm HAp. Thus, the increased biological performance of FPHAp in this study can be caused by the growth of crystal size from ~200 nm to much larger ones upon the incorporation of fluoride through calcination. In addition, our results indicating increased cell attachment are in agreement with studies on synthetic fluoride containing HAp [59]. In our previous report [30], the fluoride incorporated BAp was found to promote the proliferation and osteogenic differentiation of MG63 cells, but there was no significant difference in cell attachment on the first day, which was inconsistent with what we observed in the current study. The inconsistency in two independent studies might be due to the slight modification of sample preparation, i.e. no grinding and compressing of the materials into tablets was done before cell seeding in the current study. As surface topography plays an important role in early protein adsorption and the subsequent adhesion of cells [60], the previous work may demonstrate the effects of the fluoridated materials on osteoblastic-like cells by the slow release of fluoride ions [30], while the current study revealed the effects of fluoride ions and surface topography to be both important on biological performance of PHAp and FPHAp.

### 4.3. Solubility of PHAp/FPHAp

The degradation of calcium phosphates *in vivo* is considered to be associated with both physicochemical dissolution and osteoclast-related resorption, both of which are related to the solubility of the materials. Therefore, the solubility of these calcium phosphates should be emphasized when degradation is to be evaluated [35]. However, as extensively discussed in our earlier studies [31,32], the solubility of calcium phosphates had not been properly determined due to the complicated incongruent dissolution behavior of calcium phosphates, and some other drawbacks of the conventional methods [32,35]. In contrast, by small increments of the sample, solid titration solves the problem and achieves the exact saturation point (extremely close to the true saturation point) [31,32,34]. To avoid extra complexities, the solubility of both PHAp and FPHAp was determined in KCl solution, since the exchange between K^+^, Cl^-^ and calcium phosphate solid is believed to be ignorable [61]. Moreover, the retrograde effect on solubility with respect to increased temperature was avoided by using the constant temperature (37.0 ± 0.1°C) [33], which was also close to the physiological condition. Furthermore, considering the potential errors caused by the corrosion of glass surfaces and the subsequent precipitation due to hydrofluoric acid in the solution during the solubility determination of fluoride containing calcium phosphate, the inner surface of the reaction flask was coated with a layer of paraffin wax whose congealing point (55°C) is well above the working temperature (37°C) [40]. Lastly, the reliability of the system was reconfirmed by comparing the solubility of stoichiometric HAp with our previous reports;[32,34] the perfectly matched solubility isotherm of stoichiometric HAp between this study and our previous works indicates the solid titration system to be well calibrated.

In this study, the effect of fluoride on the solubility of apatite was found to be concentration dependent, as has been reported in fluoride substituted calcium phosphates [40,62]. This may possibly be explained by the role of fluoride in stabilizing the crystal structure of apatite. With partial fluoride substitution for hydroxyl groups, the residual hydrogen ions may bond to the incorporated fluoride ions nearby, which bear higher affinity than oxygen ions [28,63,64]. In this manner, a more stable and ‘better-ordered’ apatite structure than PHAp may be formed, with lower solubility. The effect became stronger with the increasing substitution of fluoride, corresponding to the change of the solubility isotherms (Figure 5). Besides, another speculation for the decreased solubility of FPHAp might be the formation of an FAp layer on the apatite with the presence of fluoride in the solution (undetectable as too small quantities were involved). Since the solubility of FAp was found to be lower than that of HAp [40], the presence of such an FAp layer may lower the solubility of BAp.

As a bone graft material, such low solubility of FPHAp may lead to a lower dissolution rate and a slower biodegradation process, suggesting longer resident time after implantation. This may be beneficial for some clinical indications where persistent maintenance of the bone substitute is preferred. For instance, in guided tissue regeneration, the physical support from the implanted graft material is vital for the maintenance of the augmented volume and the prevention of soft tissue collapse, which allows more time for the migration of bone cells and subsequent matrix formation. Moreover, the slow and continuous release of fluoride ion at a low concentration may be favorable for new bone formation, as has been reported in our previous study [30].

## 5. Conclusions

PHAp can be prepared from porcine bone and incorporated with fluoride via simple chemical and thermal treatments. This method contributes to the homogeneous distribution of fluoride in the material, and the distinct growth of apatite crystals. The addition of fluoride caused the difference in the shape and size of BAp crystals, increased compressive strength, decreased porosity and solubility, as well as enhanced protein adsorption and cell attachment of the apatite. The optimal level for fluoride incorporation was further demonstrated to be present in 0.25-FPHAp, manifested as its favorable mechanical property without compromising the biological performance. Therefore, a low level of fluoride incorporation could be a cost-effective way for the modification of the currently used BAp-based bone graft materials.

## Disclosure statement

No potential conflict of interest was reported by the authors.

## Funding

This work was supported by National Natural Science Foundation of China [81470783, 81400550] and the Natural Science Foundation of Guangdong Province [2015A 030311051, 2016A030310173].
